# Tuberculosis in the wild boar: Frequentist and Bayesian estimations of diagnostic test parameters when *Mycobacterium bovis* is present in wild boars but at low prevalence

**DOI:** 10.1371/journal.pone.0222661

**Published:** 2019-09-24

**Authors:** Céline Richomme, Aurélie Courcoul, Jean-Louis Moyen, Édouard Reveillaud, Oscar Maestrini, Krystel de Cruz, Antoine Drapeau, Maria Laura Boschiroli

**Affiliations:** 1 Nancy Laboratory for Rabies and Wildlife, ANSES, Malzéville, France; 2 University Paris-Est, Laboratory for Animal Health, Epidemiology Unit, ANSES, Maisons-Alfort, France; 3 Laboratoire Départemental d’Analyse et de Recherche de la Dordogne, Coulounieix-Chamiers, France; 4 Unit of Coordination and Support to Surveillance, ANSES, Maisons-Alfort, France; 5 UR 045, INRA, Corte, France; 6 University Paris-Est, Laboratory for Animal Health, Tuberculosis National Reference Laboratory, ANSES, Maisons-Alfort, France; Universitat Autònoma de Barcelona, SPAIN

## Abstract

The Eurasian wild boar (*Sus scrofa*) is increasingly considered as a relevant actor in the epidemiology of animal tuberculosis (TB). Therefore, monitoring TB in this species is key when establishing comprehensive control schemes for this disease still present in Europe. No data are available on direct and indirect TB diagnostic methods in wild boars in epidemiological contexts where TB is endemic in cattle and detected in wild boars at low prevalence. We aimed to estimate and compare sensitivity and specificity values for bacterial culture, PCR and three commercial ELISAs, i.e. the TB ELISA-VK (using the bPPD antigen), INgezim TB Porcine and IDEXX *M*. *bovis* Ab Test (both using the MPB83 and MPB70 antigens), under field conditions in France. We used frequentist methods, with bacteriology as the gold standard, and a Bayesian formulation of the latent class analysis (LCA), without using a gold standard. Submandibular lymph nodes and sera from 495 wild boars hunter-harvested in three endemic areas (Aquitaine region, Côte d’Or region, and Corsica region) were collected between 2014 and 2016. Only eight individuals were positive for *M*. *bovis* by bacteriology (1.61%; CI_95%_ 0.70–3.51%). The LCA method provided high specificities (99.2%; CI_95%_ 98.2–99.8% for INgezim TB Porcine and 99.7%; CI_95%_ 98.8–100% for IDEXX *M*. *bovis* Ab Test) and sensitivities (78.5%; CI_95%_ 65.1–88.8% for INgezim TB Porcine and 83.9%; CI_95%_ 58.9–97.2% for IDEXX *M*. *bovis* Ab Test) for both ELISAs using the MPB83 and MPB70 antigens. Bacterial culture showed limited sensitivity (42.8%; CI_95%_ 19.0–70.6%), estimated as the probability of a positive result in an animal exposed to *M*. *bovis*. PCR and ELISA using the bPPD antigens demonstrated high specificities, and sensitivities intermediates between culture and the ELISAs using the MPB83 and MPB70 antigens. These results suggest that ELISA tests using the MPB83 and MPB70 antigens are useful to detect and monitor TB exposure of wild boar populations in field conditions in France.

## Introduction

The *Mycobacterium tuberculosis* complex (MTBC) groups together mycobacterial species causing tuberculosis in humans and animals, specifically *M*. *tuberculosis*, *Mycobacterium africanum I and II*, *Mycobacterium canettii*, *Mycobacterium microti*, *Mycobacterium pinnipedii*, *Mycobacterium bovis*, and *Mycobacterium caprae*. These last two bacteria in this list cause animal tuberculosis (TB), one of the most important zoonotic diseases in Europe that is responsible for significant economic losses due to costly eradication programs and trade limitations [1].

The wild boar (*Sus scrofa*) is susceptible to TB and can become infected with the same *M*. *bovis* genotypes that infect cattle [2–7]. The role of wild boar and red deer in the epidemiology of TB varies according to different epidemiological contexts. They can be only spill-over hosts or TB-sentinels of environmental mycobacterial contamination [3,8], maintenance hosts where infection can persist without an external source [9–11], or even super-shedders excreting significantly higher amounts of tuberculous bacilli than standard shedders [8,12]. Even at low abundance, the wild boar can contribute to maintenance of TB in a host community [13], impeding TB eradication in cattle even when eradication programs have substantially reduced the incidence of TB. Diagnosing and monitoring TB in wild boars is therefore crucial in establishing comprehensive eradication schemes.

*M*. *bovis* and *M*. *caprae* are the MTBC species that have most frequently been reported in wild boars [8,14–17]. However, infection by *M*. *microti* [18–20] and non-tuberculous but potentially pathogenic environmental mycobacteria have also been reported [15,21]. The diagnosis of TB in free-ranging wildlife is relatively difficult and surveys usually rely on post-mortem examination and bacteriology. Lesions in wild boar are typically 1 mm miliary foci or caseo-calcareous tubercles of up to 5 cm in diameter located mostly in the lymph nodes of the head [14,15,22]. Bacterial culture is considered the gold standard [23] since it has the highest specificity of all available methods though it is a long process and therefore involves considerable constraints in the field. Moreover, it may produce false-negative results [24–26]. However, in the context of relatively high TB prevalence in the wild boar, the sensitivity of bacteriology has been estimated at approximately 80% [15]. An alternative to obtain faster and more sensitive diagnosis is PCR [18,24,27]. Certain serological assays have also been proposed as useful tools for screening populations and monitoring the spread of disease in Suidae because of their ease of use and generally good performances [28]. The first described used antigen was the purified protein derivative obtained from *M*. *bovis* (bovine PPD or bPPD) [29–31]. Then some studies have employed more specific antigens, such as MPB83 [32,33], MPB70 [34] and CFP10/ESAT-6 [35,36]. Recently an immunopurified subcomplex protein obtained from bPPD composed of several antigens including MPB70, MPB83, ESAT-6 and CFP-10, named P22, has also been used [28,37].

The aim of this study was to compare five direct and indirect diagnostic tests for TB in hunter-harvested wild boars in areas in France where TB is persistent at low prevalence rates in cattle and reported at a low frequency in wild boars [7]. We used frequentist methods to estimate the sensitivity, specificity, positive predictive value, and negative predictive value of PCR and three different commercial ELISA tests, with bacteriology as the gold standard. We also used Bayesian latent class analysis (LCA) to estimate and compare the sensitivities and specificities of these five diagnostic tests, in the absence of a reference test using latent class analysis.

## Materials and methods

### Ethics statement

All samples were collected from wild boars legally hunted for recreational purposes. This study did not involve deliberate killing of animals. No ethical approval was deemed necessary.

### Collection of samples

A sampling design was implemented to collect samples from hunter-harvested wild boars during the 2014–2015 and 2015–2016 hunting seasons in four different populations of wild boars from TB endemic areas in France: Dordogne, Pyrénées-Atlantiques,—both in the Nouvelle-Aquitaine region in the South-West -, Côte d’Or—in the Center-East -, and the island of Corsica in the Mediterranean sea.

Specifically for the present study, submandibular lymph nodes were removed from each studied animal in laboratories in Aquitaine and Côte d’Or as part of the French surveillance program for TB in wildlife [7], and in Corsica in the field. On the same individuals, thoracic blood samples were collected by hunters a maximum of 8 hours after death. The presence of gross TB-like lesions on submandibular lymph nodes or any organ was reported on the field by trained hunters [38] or veterinarians when doing the post-mortem examination or at the local laboratory.

Lymph nodes were kept frozen at -20°C until bacteriology and extraction of DNA for PCR. Serum was removed by centrifuge within 24h, and serum samples were frozen at -20°C until serologic tests were performed.

Only one animal was analysed only by PCR but neither by culture nor by serology.

### Diagnostic tests

#### Bacteriology

Culture was performed on the submandibular lymph nodes, whether or not presenting lesions, following the protocol established by the French National Reference Laboratory (NF U 47–104) for isolation of MTBC. After decontamination, the supernatant was seeded on two different media: Löwenstein-Jensen and Coletsos [39]. All seeded media were incubated at 37°C ±3°C for 3 months and examined every 2 weeks. Mycobacterial isolates were identified by DNA amplification [40]. Briefly, mycobacterial thermolysates were analyzed by real-time PCR targeting insertion sequences IS*6110* and IS*1081* for MTBC identification, IS*1245* for *Mycobacterium avium* complex (MAC) identification, and the 65 kDa heat shock protein gene (hsp65) for *Mycobacterium* spp. detection (S1 Table). Real-time PCR assays were performed in a final volume of 25 μL using the TaqMan Fast Universal PCR Master Mix (Roche Diagnostics, Meylan, France) at a 1X final concentration, with primers at 300 nM and probes at 250 nM. PCR cycling consisted of 2 min at 50°C and 20 s at 95°C, followed by 50 cycles of 2-step amplification with 3 s at 95°C, and 30 s at 60°C.

MTBC complex species were confirmed and identified by Luminex spoligotyping [41].

A result was classified as positive when *M*. *bovis* were cultured, while a negative result was recorded when no organisms were isolated or when other *Mycobacterium* species (*Mycobacterium avium* and other non-tuberculous mycobacteria (NTM)) were cultured.

#### Tuberculosis PCRs

A commercial kit was used: LSI VetMAXTM *Mycobacterium tuberculosis* Complex PCR Kit, 2 wells. The targeted sequence was IS*6110*, which is present in all species of the *M*. *tuberculosis* complex [42]. After mechanical lysis of tissue, DNA was extracted using a QIAamp DNA mini kit (Qiagen, Germany) or MagVet Universal Isolation Kit MV384 (Thermo Fisher Scientific, USA) with a King Fisher KF96 automate (Thermo Fisher Scientific, USA), following the manufacturer’s instructions. Then, 5 μL of the extracted DNA was mixed with 20 mL of reaction mix and the reaction was carried out at 50°C for 2 minutes (1 cycle), followed by one cycle of 10 min at 95°C, and 40 cycles of 15 s at 95°C and 1 min at 60°C. Results were interpreted as negative, positive (CT≤38) or invalidated, following the manufacturer’s recommendations and by comparison with negative and positive controls.

Positive DNA was further characterized to determine the species within the MTBC by Luminex spoligotyping [41].

#### Serological tests

Serum samples, which had previously gone through fewer than five freeze/thaw cycles [43], were tested by means of three indirect ELISAs: one that used bPPD, TB ELISA-VK (Vacunek S.L, Derio, Spain) (bPPD1 ELISA; cut-off 0.2 [29]–and 0.5 [30]), and two using recombinant MPB83 and MPB70 proteins as coating antigens, INgezim TB Porcine (Ingenasa, Madrid, Spain) (cut-off 0.3; manufacturer’s instructions) and IDEXX *M*. *bovis* Ab Test (IDEXX Laboratories) (cut-off 0.3; manufacturer’s instructions). This last kit commercialised for bovine has been adapted for the present study using porcine conjugate.

### Data analysis

#### Frequentist analysis

Apparent TB prevalence (P) was calculated as the number of animals with *M*. *bovis* isolation in culture, divided by the number of tested animals. The 95% confidence interval (CI_95%_) was calculated using exact binomial tests.

Sensitivity (Se) and specificity (Sp) estimates with CI_95%_ calculated by the Clopper-Pearson method, positive predictive values (PPVs), negative predictive values (NPVs), and test agreement between serological assays, calculated as Cohen’s kappa coefficient, were estimated using Epitools (Ausvet Pty Ltd, Australia http://epitools.ausvet.com.au).

#### Bayesian analysis

Two analyses were carried out using a Bayesian formulation of the latent class model. With this approach, the available tests allowed us to classify animals into one of two latent classes: i) wild boars that have previously been exposed to *M*. *bovis* (current or past TB infection), and ii) wild boars that have never been exposed to *M*. *bovis*. We used the results of TB surveillance in wild boars from the 2013–2014 hunting campaign (Sylvatub data) to define three wild boar populations with differing expected animal exposure prevalences: the Dordogne population, the Cote d’Or population, and the Corsica population. Tests results from Pyrénées-Atlantiques were not taken into account as only a few animals were hunted there.

The first analysis aimed to assess the sensitivity and specificity of the five diagnostic tests (bacteriology, IDEXX *M*. *bovis* Ab Test, INgezim TB Porcine, PCR, and TB ELISA-VK cut-off 0.5), as well as the prevalence of wild boar exposure to *M*. *bovis* in Dordogne and Corsica from the animal tests results in these two populations (the wild boar samples from Côte d’Or were not subjected to the five tests). The second analysis aimed to assess the sensitivity and specificity of bacteriology, the IDEXX *M*. *bovis* Ab Test, INgezim TB Porcine, and PCR, as well as the prevalence of wild boar exposure to *M*. *bovis* in the three study areas (Côte d’Or, Dordogne and Corsica) on the basis of the test results for the animals from these three populations that were subjected to the four diagnostic tests.

As the IDEXX *M*. *bovis* Ab Test and INgezim TB Porcine detect the same antigens, we assumed conditional dependence of test sensitivities and specificities. Bacteriology and PCR were also considered dependent tests given the exposure status, as they both detect *M*. *bovis* directly. Three additional parameters were then assessed in both analyses: covariances between i) the sensitivities of the two serological tests, ii) the specificities of the two serological tests, and iii) the sensitivities of bacteriology and PCR. As the specificity of bacteriology was fixed at 1 in both analyses, the covariance between the specificities of bacteriology and PCR was assumed to be null.

For all the parameters to be estimated, except for the sensitivity of INgezim TB Porcine, we used uninformative priors, in the shape of a distribution on the interval between 0 and 1 (modeled using the beta (1, 1) distribution) except for the specificities of PCR and TB ELISA-VK and the covariances (see hereafter). As the sensitivity of INgezim TB Porcine has recently been assessed at 78.0% [CI_95%_: 65.3–87.7] on 147 pigs from South-Eastern Spain [34], we used an informative prior for this parameter with a mean of 0.78 and a 2.5^th^—97.5^th^ percentile range of 0.631–0.899 (modeled using a beta (27.3, 7.7) distribution). The IDEXX *M*. *bovis* Ab Test and INgezim TB Porcine specifically detect *M*. *bovis*, while PCR and TB ELISA-VK only detect the MTBC. We therefore assumed that the specificities of PCR and TB ELISA-VK were lower than those of the IDEXX *M*. *bovis* Ab Test and INgezim TB Porcine: for their priors, we used a uniform distribution between 0 and the minimum value between the specificities of the IDEXX *M*. *bovis* Ab Test and INgezim TB Porcine. The specificity of bacteriology was fixed to 1. For each covariance, we used as a prior a uniform distribution between 0 and the maximum value of this covariance (computed function of the sensitivities (or specificities) of the two dependent tests).

The analyses were implemented with RStudio, version 1.1.383 (http://www.rstudio.com) and the R2OpenBUGS package [44]. For each analysis, 40 000 iterations with a thin interval of two were performed. The first 20 000 iterations were discarded as burn-in, and two chains were run from different initial values. To assess the convergence of these chains, we visually checked the Gelman–Rubin diagnostic plots. Supporting information on raw commands coding the models (without annotation) of the latent class analysis can be found in S1 and S2 Texts.

## Results

**Tissue samples and sera** were collected from 495 hunter-killed wild boars from the 4 study areas (Table 1): 128 in Corsica, 223 in Côte d’Or, 123 in Dordogne, and 21 in Pyrénées-Atlantiques. The lymph nodes that did not show gross TB-like lesions collected in Côte d’Or in 2014–2015 unfortunately did not undergo PCR analysis in the laboratory because of internal reasons. A second sampling campaign in 2015–2016 made it possible to collect 112 other wild boar samples, which were analyzed in culture, PCR and via serology with INgezim TB Porcine and the IDEXX *M*. *bovis* Ab Test, but not with TB ELISA-VK, as the kit was no longer available.

**Table 1:** Number of animals sampled and tested by sampling area and hunting season.

Sampling area (hunting season)		Wild boar sampled	Sera	Lymph nodes analyzed by bacteriological culture	Lymph nodes analyzed by PCR
**Côte d’Or**	(2014–2015 ^(1)^)	111	111	111	2
	(2015–2016)	112	112 ^(2)^	112	112
**Dordogne** (2014–2015)		123	123	123	123
**Pyrénées-Atlantiques** (2014–2015)		21	21	21	21
**Corse** (2014–2015)		128	128	128	128
**Total**		495	495	495	386

^(1)^ The lymph nodes that did not show gross TB-like lesions collected in Côte d’Or in 2014–2015 did not undergo PCR analysis.

^(2)^ Serology with TB ELISA-VK was not performed.

Gross lesions were reported in samples from 17 individuals of the 495 wild boars.

**Bacteriological culture** of samples from the 495 wild boars showed 8 animals to be *M*. *bovis* positive by culture (*p* = 1.61%; CI_95%_ 0.70–3.51%): 1/128 in Corsica (*p* = 0.8%, CI_95%_ 0.01–4.3%), 3/223 in Côte d’Or (2/111 in 2014–2015, *p* = 1.8%, CI_95%_ 0.2–6.4%, and 1/112 in 2015–2016, *p* = 0.9%, CI_95%_ 0.0–4.9), and 4/123 (3.3%—CI_95%_: 0.8–8.2) in Dordogne. No *M*. *bovis* was isolated from the 21 samples from the Pyrénées-Atlantiques Landes. Except for *M*. *bovis*, no other species from the MTBC were detected by culture. MAC was isolated from 10 animals, and NTM from 14 animals. Five of the eight *M*. *bovis*-positive individuals had gross lesions.

Of the 386 wild boars that were tested by **PCR**, 17 were positive for MTBC (4.4%; CI_95%_ 2.6–6.9), 8 for which *M*. *bovis* was confirmed by spoligotyping (of which 4 were bacteriology positive and had gross lesions, and one bacteriology negative but with gross lesions), but 9 could not be identified within the MTBC. All of these 9 wild boars presented gross lesions but were negative by bacteriology.

**Serological analyses** showed (i) for TB ELISA-VK, 53 seropositive out of 382 serum samples available considering the 0.2 cut-off (recommended by the manufacturer) (13.9%, CI_95%_ 10.6–17.7%), and 16 seropositive out of 382 considering the 0.5 cut-off (which maximizes specificity) (4.2%, CI_95%_ 2.4–6.7%), (ii) for the IDEXX *M*. *bovis* Ab Test, 23 seropositive wild boars out of 494 (4.7%, CI_95%_ 2.9–6.9%), and (iii) for INgezim TB Porcine, 22 out of 494 (4.5%, CI_95%_ 2.8–6.6%) (Table 2). Fifteen wild boars were seropositive for the three ELISAs when using the cut-off 0.2 for TB ELISA-VK (13 if cut-off was 0.5), and 326 seronegative in three ELISAs. 2x2 comparative results between the tests are presented in Table 2. For details on positive wild boars for at least one of the tests, see S2 Table

**Table 2:** Comparative results between ELISAs (P: Seropositive, N: Seronegative).

		IDEXX *M*. *bovis* Ab Test		INgezim TB Porcine		TB ELISA-VK		TB ELISA-VK	
						Cut-off 0.2		Cut-off 0.5	
		P	N	P	N	P	N	P	N
		23	471	22	472	53	329	16	367
IDEXX *M*. *bovis* Ab Test	P			20	3	15	1	13	4
	N			2	469	38	326	3	361
INgezim TB Porcine	P	20	2			15	3	13	5
	N	3	469			38	326	3	362
TB ELISA-VK	P	15	38	15	38			16	37
Cut-off 0.2	N	1	326	3	326			0	329

The agreement between the two tests using the MPB80 and MPB73 antigens, the IDEXX *M*. *bovis* Ab Test and INgezim TB Porcine, was found to be excellent (k = 0.88, CI_95%_ 0.78–0.98). The agreement of these two tests with TB ELISA-VK was found to be good when using the 0.5 cut-off (k = 0.78, CI_95%_ 0.62–0.94, and k = 0.75 [0.59–0.92], respectively), but poor when using the 0.2 cut-off (k = 0.39, CI_95%_ 0.25–0.54, and 0.38, CI_95%_ 0.23–0.52, respectively).

The parameters of the diagnostic tests estimated by the frequentist method are shown in Table 3.

**Table 3:** Sensitivity, specificity, PPV (Positive predictive value) and NPV (negative predictive value) of PCR and ELISAs with culture as the gold standard.

Diagnostic test	Sensitivity (CI_95%_)	Specificity (CI_95%_)	PPV	NPV
PCR	62.5% (24.6–91.5)	97.1% (94.8–98.5)	0.31	0.99
IDEXX *M*. *bovis* Ab Test	75% (34.9–96.8)	96.7 (94.7–98.1)	0.27	0.99
INgezim TB Porcine	75% (34.9–96.8)	96.9% (94.9–98.3)	0.29	0.99
TB ELISA-VK cut-off 0.2	85.7% (42.1–99.6)	87.5% (83.7–90.6)	0.11	0.99
TB ELISA-VK cut-off 0.5	85.7% (42.1–99.6)	97.3% (95.2–98.7)	0.38	0.99

**Fig 1:**
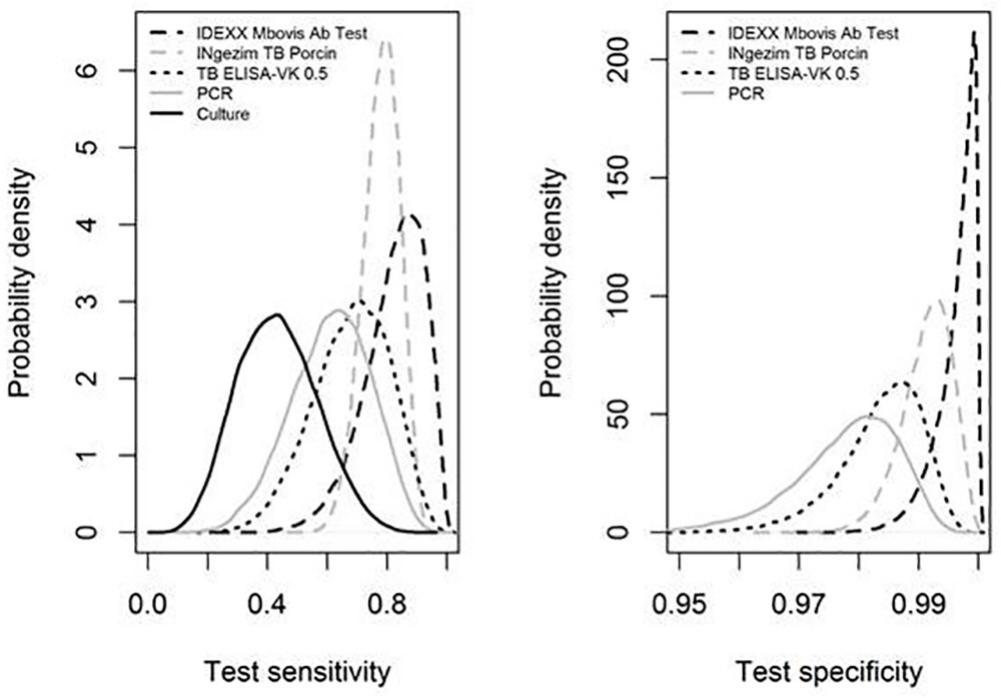
Results of the latent class analysis A: *a posteriori* distribution of sensitivities (a) and specificities (b) of the 5 tests. The specificity of the culture was not estimated because set at 100%.

The first **Bayesian analysis** A, based on the results from 251 wild boars sampled in Corsica and Dordogne, shows broad posterior distributions of sensitivities except for INgezim TB Porcine (given the use of an informative prior), which overlap to a large extent (Table 4 and Fig 1). It is therefore not possible to determine which test is the most sensitive. However, bacterial culture appears to be less sensitive than the IDEXX *M*. *bovis* Ab Test and INgezim TB Porcine. The specificities of all the tests are high (between 95% and 100%). The *a posteriori* distributions also largely overlap: even though the IDEXX *M*. *bovis* Ab Test seems more specific than PCR and TB ELISA-VK, it is not possible to conclude whether it is more specific than INgezim TB Porcine.

**Table 4:** Prevalence in sampling areas, sensitivity and specificity of the 5 tests estimated by the LCA—Analysis A.

Parameters	Median	2.5th percentile	97.5th percentile
Prevalence in Corsica	2.1%	0.5%	5.7%
Prevalence in Dordogne	8.2%	4.1%	14.1%
Se IDEXX *M*. *bovis* Ab Test	83.9%	58.9%	97.2%
Sp IDEXX *M*. *bovis* Ab Test	99.7%	98.8%	100%
Se INgezim TB Porcine	78.5%	65.1%	88.8%
Sp INgezim TB Porcine	99.2%	98.2%	99.8%
Se TB ELISA-VK cut-off 0.5	69.0%	41.2%	90%
Sp TB ELISA-VK cut-off 0.5	98.5%	96.7%	99.4%
Se PCR	61.9%	33.5%	84.9%
Sp PCR	97.9%	95.6%	99.1%
Se culture	42.8%	19%	70.6%
Covariance Se IDEXX *M*. *bovis* Ab Test /IINgezim TB Porcine	0.066	0.006	0.16
Covariance Se PCR/culture	0.032	0.001	0.114
Covariance Sp IDEXX *M*. *bovis* Ab Test /INgezim TB Porcine	0.001	0	0.006

**Fig 2:**
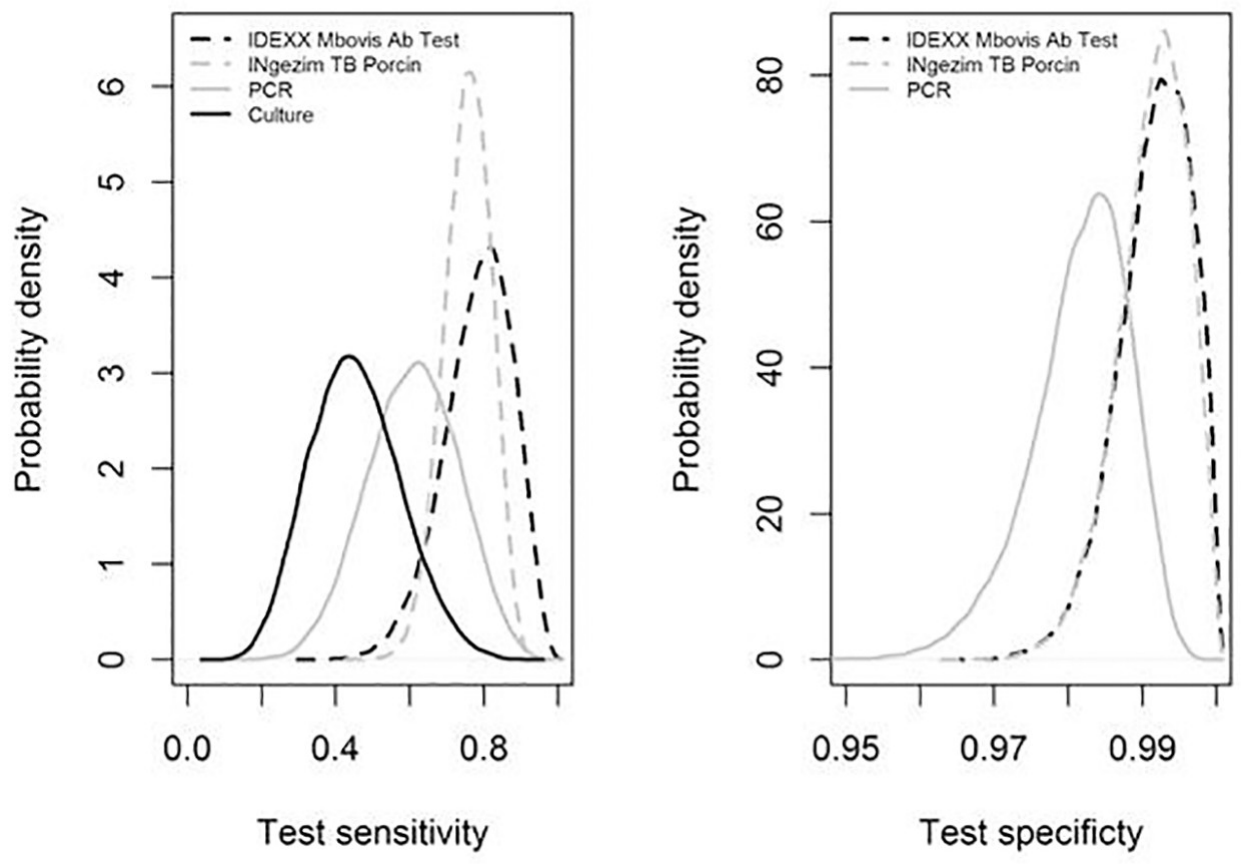
Results of the latent class analysis B: a posteriori distribution of sensitivities (a) and specificities (b) of 4 tests. The specificity of the culture was not estimated because set at 100%.

The second analysis B, performed on the results for 362 wild boars sampled in Corsica, Dordogne and Côte d’Or, shows equivalent sensitivities for the IDEXX *M*. *bovis* Ab Test and INgezim TB Porcine, which are good (median 75–80%), and high specificity for these two tests (median greater than 99%) (Table 5 and Fig 2). Both serological tests appear to be more sensitive than the bacterial culture. Since posterior distributions of sensitivities and specificities of the different tests overlap widely, we cannot conclude on the characteristics of PCR, which remains a very specific test (median of 98%), but with sensitivity that is more difficult to estimate, and likely intermediate between the sensitivity of bacterial culture and that of serological tests.

**Table 5:** Prevalence in sampling areas, sensitivity and specificity of the 4 tests estimated by LCA—Analysis B.

Parameters	Median	2.5th percentile	97.5th percentile
Prevalence in Corsica	1.9%	0.3%	5.7%
Prevalence in Côte d’Or	5.5%	2.1%	11.3%
Prevalence in Dordogne	8.7%	4.3%	15.2%
Se IDEXX *M*. *bovis* Ab Test	79.3%	57.5%	93.8%
Sp IDEXX *M*. *bovis* Ab Test	99.2%	98%	99.9%
Se INgezim TB Porcine	75.8%	62.3%	86.9%
Sp INgezim TB Porcine	99.2%	98%	99.9%
Se PCR	61.1%	36.3%	83.8%
Sp PCR	98.2%	96.6%	99.2%
Se culture	44.7%	23.1%	70.7%
Covariance Se IDEXX *M*. *bovis* Ab Test /IINgezim TB Porcine	0.096	0.019	0.178
Covariance Se PCR/culture	0.032	0.001	0.111
Covariance Sp IDEXX *M*. *bovis* Ab Test /INgezim TB Porcine	0.002	0	0.01

## Discussion

We aimed to determine and compare the characteristics of direct and indirect TB diagnostic tests in free-living wild boars under field conditions in France. The first noteworthy result of this study was the low apparent prevalence of infection estimated by bacterial culture within each of the areas (ranging from 1.9% to 8.7% when estimated by LCA). In Europe, the prevalence of TB in wild boars differs among countries and even within regions: for instance, it is low in some areas of Italy (3%) [3] or Portugal (around 10%) [15,45], and high in some regions of Spain, especially in the South Central area, ranging from 52% to 70%, or even near 100% locally in some populations [9,46]. In France, low prevalence was expected in Dordogne and in Côte d’Or, where previous results obtained in the framework of the national surveillance program Sylvatub were similar [7]. In Corsica we expected a slightly higher infection rate in wild boars compared to previous data in wildlife [47]. Moreover, for the present study, we targeted on the North-East of the island, an area with previous outbreaks in cattle [48] and cases in domestic swine [47], and where interspecific contacts, especially on waste dumpsites, are common.

The main limitation of our study is the small number of infected wild boars found by bacteriology, which is probably the consequence of the very low prevalence of the disease in our population. This limitation may lead to significant imprecision in estimating the sensitivities of the employed serological tests by a frequentist analysis. It also has considerable impact on the positive predictive values of the tests (lower positive predictive value at low prevalence), which can therefore only be transposed to similar contexts with low prevalence.

Another diagnostic result that impedes full analysis of our data is that positive PCR DNAs from nine wild boars remained inconclusive by spoligotyping, and we therefore could not elucidate which MTBC elicited a positive PCR. This result is sometimes observed in other species, domestic (cattle) or wild (badgers), or even in wild boars (M. L. Boschiroli, pers. comm.). However, in the present study, this concerns more than half of the positive PCRs and implies that, contrary to what we expected, we could not use molecular analysis to classify individuals as infected or not by *M*. *bovis*, nor to better estimate the characteristics of the serological tests to *M*. *bovis* exposure. In fact, wild boars for which the PCR was positive to MTBC could have been infected by *M*. *microti*, which is widely present in some of the studied areas and difficult to isolate by bacteriology [20]. We can, however, note that four wild boars negative in bacteriology, positive in MTBC-PCR, but with inconclusive spoligotyping were positive in serology with the tests using MPB83 and MPB70. Because these antigens are major antigens of *M*. *bovis* [49], we can hypothesize that detected DNA was from *M*. *bovis*.

When using frequency analysis for estimation, the test with the best characteristics with regard to culture was TB ELISA-VK interpreted at the 0.5 threshold, a threshold which maximizes specificity [29]. The use of this test in a previous study had shown promising results for surveillance of animal tuberculosis in wild boar populations in France [30]. One of the questions raised in this previous study was whether some seropositive results might be due to exposure to MTBC bacteria, such as *M*. *microti*, and not *M*. *bovis*. One of the reasons for having included samples from Corsica was that the circulation of *M*. *microti* is expected to be at low prevalence, or even non-existent there. The presence of *M*. *microti* in European Mediterranean ecosystems, such as in Spain has yet not been reported in wild or domestic animals, contrary to temperate ecosystems [20]. The results obtained in Corsica do not shed any further light on this question since there are too few positive wild boars by culture, PCR or serological analysis. It should also be noted that this kit requires a stage of plate coating with the bPPD antigen, which introduces a critical additional step to the test and could be a source of lack of intra and inter-laboratory reproducibility.

The purpose of the latent class analysis in a Bayesian framework was to estimate the intrinsic characteristics of direct and indirect diagnostic tests used in wild boars, without designating a reference test. These estimates were made by considering as positive ("sick" latent condition) a wild boar that had been exposed to *M*. *bovis*, whether or not it was still infected, and as negative ("not sick") a wild boar that had never been exposed to *M*. *bovis*. As for frequency analysis, estimates were found to be imprecise (large credibility intervals). For latent class models, at least two populations with different prevalence values are needed, and, according to Toft et al. [50], it is preferable to have as large a difference in prevalence as possible between these populations. In our case, based on previous results, we expected two or three populations with relatively different prevalence values (Corsica, Côte d’Or and Dordogne) [38]. However, the prevalence observed in the present study proved to be similar among the different regions, which reduces the precision of the estimates as mentioned above. It could even have biased the estimates themselves (the most contrasting situations being between Corsica and Dordogne, and conversely to what we expected). In this context, it was not possible to classify the tests according to their sensitivities and specificities.

However, three groups of tests stand out. Firstly, bacterial culture with perfect specificity but limited sensitivity, the sensitivity here being the probability of giving a positive result in an animal previously exposed to *M*. *bovis*. Secondly, both serological tests using the MPB83 and MPB70 antigens (the IDEXX *M*. *bovis* Ab Test and INgezim TB Porcine) with very good specificity and sensitivity. Thirdly, PCR and TB ELISA-VK at the 0.5 threshold with very good specificity, but sensitivity likely to be intermediate between those of the first two groups, although this sensitivity is difficult to estimate with the present data set.

The IDEXX *M*. *bovis* Ab Test is a kit marketed for use in cattle. This test was used here after adaptation of the kit using a porcine conjugate. The only kit currently on the market for suids is the INgezim TB PORCINE test. Its characteristics were similar to those of the IDEXX *M*. *bovis* Ab Test under the conditions of our study, with agreement between tests (k = 0.88) considered excellent: good sensitivity to *M*. *bovis*, even if estimated in a relatively imprecise manner (by frequentist analysis: 75% [34.9–96.8]); in Bayesian analysis: 75.8% [62.3–86.9]) and excellent specificity (97% [94.9–98.3]); 99.2% [98–99.9]).

## Conclusion

On the basis of our findings, and despite all the precautions for interpretation mentioned above because of the low prevalence context, we suggest that in and around areas where cattle or wildlife are infected, ELISAs using MPB83 and MPB70 could be used in France for wild boar population monitoring. This would make it possible to test a large number of individuals, with simpler logistics and much lower cost compared to current surveillance, which relies on the sampling of hunter-harvested wild boar heads, sent to the nearest local laboratory for PCR on submandibular lymph nodes. This serological surveillance would allow for temporal monitoring of the circulation of *M*. *bovis* in wild boar populations in areas where the infection is already detected in this species, and where the aim is not to identify TB-infected wild boars, but rather to assess changes in TB prevalence in wild boar populations. In areas where the infection has not yet been reported in wild boars, being only detected in cattle or captive wild boars, the detection of seropositive free-living wild boars should prompt research on TB infection using direct diagnosis (PCR and/or culture) implemented at a finer scale in the area concerned by seropositivity in wild boars.

## Supporting information

S1 Table Oligonucleotide primers and probes for real-time-PCR assays used in this study. (XLSX)

S2 Table Results of culture, PCR and serological tests of wild boar samples positive for at least one of the tests. (XLSX)

S1 Text Supporting information on raw commands coding the models without annotation of the latent class analysis. (TXT)

S2 Text Supporting information on raw commands coding the models without annotation of the latent class analysis. (TXT)
